# Predicting Lexical Priming Effects from Distributional Semantic Similarities: A Replication with Extension

**DOI:** 10.3389/fpsyg.2016.01646

**Published:** 2016-10-24

**Authors:** Fritz Günther, Carolin Dudschig, Barbara Kaup

**Affiliations:** Department of Psychology, University of TübingenTübingen, Germany

**Keywords:** Latent Semantic Analysis, distributional semantics models, semantic priming, associative priming

## Abstract

In two experiments, we attempted to replicate and extend findings by Günther et al. (2016) that word similarity measures obtained from distributional semantics models—Latent Semantic Analysis (LSA) and Hyperspace Analog to Language (HAL)—predict lexical priming effects. To this end, we used the pseudo-random method to generate item material while systematically controlling for word similarities introduced by Günther et al. (2016) which was based on LSA cosine similarities (Experiment 1) and HAL cosine similarities (Experiment 2). Extending the original study, we used semantic spaces created from far larger corpora, and implemented several additional methodological improvements. In Experiment 1, we only found a significant effect of HAL cosines on lexical decision times, while we found significant effects for both LSA and HAL cosines in Experiment 2. As further supported by an analysis of the pooled data from both experiments, this indicates that HAL cosines are a better predictor of priming effects than LSA cosines. Taken together, the results replicate the finding that priming effects can be predicted from distributional semantic similarity measures.

For speakers of a language, it is quite intuitive that some words, such as *tiger* and *lion* or *tiger* and *cage*, are more similar in meaning than other words, say *tiger* and *rainbow*. One possibility for this is that in such cases, the concepts denoted by the words are *semantically related*. Two concepts that are semantically related can belong to the same superordinate category, in which case they typically share similar features or properties (as is the case for *tiger* and *lion*, which are both *cats* and therefore both have *paws, teeth* and a *tail*, and are *carnivores*); one of the concepts can be a category that includes all instances of the other concept (for example, every *tiger* is a *cat*, but not every *cat* is a *tiger*); or one of the concepts can be a part belonging to the other concept (a *paw* is a part of a *tiger*).

On the other hand, words can also be similar if they often co-occur in the same context, even without having a semantic relation, as is for example the case for *tiger* and *cage*. In cases such as these, the respective concepts are *associatively related*. As an example, Moss et al. (1994) define associative relations between words as frequent local co-occurrences (i.e., within a window of several words).

Such word similarities (we use this term to refer to similarities *in meaning*, as opposed to for example phonetic similarities) play a major role in the psychology of language, psycholinguistics, and cognitive science. On the one hand, they are important in theoretical models, such as models of language comprehension (e.g., Kintsch, 1988, 2001) or the mental lexicon (e.g., Quillian, 1967; Collins and Loftus, 1975). On the other hand, they often are a variable of interest or control variable in experimental studies relying on language material (e.g., Van Petten, 2014; Dudschig et al., 2016; Nieuwland, 2016).

Several methods have been developed to empirically assess word similarities. One of the most commonly employed methods are subjective similarity ratings, where word pairs are presented to participants, and they are asked to indicate on a given scale how similar these words are (Rubenstein and Goodenough, 1965; Miller and Charles, 1991; Finkelstein et al., 2001). Another method is the collection of free association norms (Nelson et al., 2004), where one word is presented to participants, and they are asked for the first meaningfully related or strongly associated word that comes to their mind. The relative frequency of a given answer is then taken as a measure of word similarity.

## Distributional models of semantics

Another approach which emerged in computational linguistics is to assess word similarities not from human participants, but instead from large amounts of text. A prominent class of models incorporating this approach are *distributional semantics models* (DSMs), which rely on the *distributional hypothesis* that words with similar meanings tend to occur in similar contexts (Harris, 1954). In DSMs, word meanings are represented as high-dimensional numerical vectors. These vectors are constructed by counting the co-occurrences of words with pre-defined contexts—these co-occurrence counts already constitute the vectors, if the order of contexts is the same for all words—and applying statistical routines to those co-occurrence counts. These routines include weightings to reduce the impact of word frequencies (Church and Hanks, 1990; Martin and Berry, 2007) and dimensionality reduction techniques to get rid of noise and to identify more basic semantic dimensions (Landauer and Dumais, 1997; Martin and Berry, 2007; Dinu and Lapata, 2010). With such vector representations, it is possible to compute word similarities using geometrical measures; the most commonly used measure is the *cosine similarity*, defined as the cosine of the angle between two vectors. An advantage DSMs have over participant-based techniques, such as similarity ratings or free associations, is the possibility to obtain word similarities for large sets of word pairs without investing huge amounts of resources and effort.

Highly prominent DSMs, especially in cognitive science, are Latent Semantic Analysis (LSA, Deerwester et al., 1990; Landauer and Dumais, 1997) and the Hyperspace Analog to Language (HAL, Lund and Burgess, 1996). The main difference between those models lies in the definition of *context* they rely on. In LSA, a context is defined as the document a word occurs in. A document is defined as a collection of words, and can be a sentence, a paragraph, or an article, for example. Therefore, the co-occurrence count matrix in LSA is a word × document matrix. In HAL, on the other hand, the context of a word is defined as the words within a given window around it (for example, the three words to its left and its right). The co-occurrence count matrix in HAL is therefore a word × word matrix.

The different definitions of context result in different vector representations, which capture different kinds of information. As pointed out by Sahlgren (2008), LSA focusses on *syntagmatic* relations between words (i.e., which words occur together?), while HAL focusses on *paradigmatic* relations (i.e., which words can be replaced by one another?). On the concept level, these relations correspond to associative and semantic relations, respectively. Empirical results supporting this argument come from Jones et al. (2006), who showed that LSA cosine similarities are better at predicting associative priming effects, while HAL cosine similarities are better at predicting semantic priming effects.

In the recent years, a series of other DSMs which focus on different aspects of language and the cognitive system have been developed. For example, the BEAGLE model is based on both word-based and document-based co-occurrences of words (Jones and Mewhort, 2007), and is designed to simultaneously capture associative and semantic relations between words. This model starts with initial random vectors of fixed dimensionality, that get updated with every document in the corpus. The StruDEL model (Baroni et al., 2010) is designed to capture properties of concepts. It takes as input a given list of concepts with annotated properties (such as “father OF children”), as well as a corpus. As an output, it can estimate how likely it is that two given concept are linked by a specific relation (such as FOR or OF). Topic Models (Griffiths et al., 2007) represent words as a probability distribution over topics, and are, similar to LSA, derived from word-by-document count matrices. On the other hand, there are also other models derived from word-by-word co-occurrence matrices, similar to HAL, such as the *Contextual Self-organizing Map* (SOM) model by Zhao et al. (2011), which aims at clustering words with similar meanings together in a semantic space. More recently, prediction-based models such as *word2vec* (Mikolov et al., 2013) have been developed that do not rely on counting co-occurrences between words in a text corpus, but rather tune a word vector to best predict its surrounding context words, or to be best predicted by them. All these models have been shown to give good results in selected tasks; however, it is out of the scope of this article to analyse similarity measures from all these models. Instead, we will focus on HAL and LSA in our experiments.

Notably, such distributional vectors are collected purely from text data. Therefore, DSM similarities do not *necessarily* reflect psychological word similarities (Sahlgren, 2008); the question whether they do or not is therefore an empirical one. This question is indeed important, since word similarities are widely used in language psychology and psycholinguistics, and DSMs offer a very convenient and economic method of obtaining them. If these similarities are to be used in psychological theories and studies, however, it is crucial that the employed word similarity measures represent a psychologically plausible variable.

## Distributional semantics and priming

This issue of the psychological validity of DSM similarities has already been addressed in several studies using lexical priming techniques (introduced by Meyer and Schvaneveldt, 1971). In lexical priming studies, two words are presented successively, and participants have to react to the second word, the *target* (for example in lexical decision tasks or naming tasks). The general assumption is that such a response is facilitated and therefore faster if the two words have similar meanings, both for associative and semantic similarity (Ferrand and New, 2003; Hutchison, 2003).

In an early study on the relation between DSM similarities and priming, Lund et al. (1995) employed three experimental conditions in a priming experiment—in one condition, primes and targets were only semantically related, in a second condition they were only associatively related, and in a third condition there was a semantic as well as an associative relation. Priming effects were observed in the two conditions including semantically related word pairs. These authors then computed the similarity between the primes and the targets in all three conditions, and only found a significant difference in HAL similarities for the conditions including semantic relations between primes and targets, but not for the purely associatively related pairs. Similar analyses based on existing priming data (Hodgson, 1991) have also been made by Padó and Lapata (2007), who found differences in DSM similarities between experimental conditions including (semantically and associatively) related and unrelated prime-target pairs that also showed semantic priming effects. Lund and Burgess (1996) also re-analyzed semantic priming data from Chiarello et al. (1990) and found that HAL similarities were significantly correlated with semantic priming effects in this study.

In an extensive re-analysis of the data from several priming experiments with semantically as well as associatively related word pairs, Jones et al. (2006) found that LSA and HAL cosine similarities predicted priming effects. They observed the pattern that LSA was better at predicting associative priming effects, while HAL was better at predicting semantic priming effects. Very recently, Mandera et al. (2017) analyzed a very large data set of semantic priming (Hutchison et al., 2013) with similarity measures derived from a count-based HAL model, and prediction-based DSMs (Mikolov et al., 2013). They found that all of these models predicted priming effects to a quite high degree, with an optimally parameterized count-based model performing equally well as the prediction-based models.

In an experimental study, Hutchison et al. (2008) found that LSA cosine similarities were higher in a set of related word pairs than they were for unrelated word pairs, while at the same time reaction times were faster for the related pairs. However, while they found these group differences, LSA cosines did not predict reaction times at the single item level within those groups.

Critically, in all the studies cited here, DSM similarities were always analyzed as a *post-hoc* variable on an existing data set of priming. Furthermore, only studies were analyzed in which a priming effect had already been observed, and it was then checked whether there was also a difference between related and unrelated word pairs in terms of DSM similarities. This approach has several drawbacks; for example, data sets with DSM similarity differences between unrelated and related pairs that do *not* show priming effects have no chance to end up in such analyses. We therefore argue to start the empirical investigation from the DSM similarity measures, and not from existing priming effects. To this end, it is necessary to directly manipulate LSA cosine similarities as the independent variable of interest.

We argue that this is an important empirical test, providing insight that the *post-hoc* analyses of already observed priming effects discussed above cannot deliver. In such analyses, the only negative result that can occur is that one has a priming effect, but no difference in DSM similarities between the items that cause the effect. In our experiments, it is possible to observe the negative result that there is no priming effect, despite the difference in DSM similarities between items. The first type of analysis can therefore only be employed to address the issue whether an observed priming effect always implies a difference in DSM similarities (i.e., whether differences in DSM similarities are necessary condition for priming effects). However, in order to investigate whether differences in DSM similarities are a *sufficient* to produce priming effects, one has to start from these similarities. To our knowledge, this issue has been neglected in the literature.

We recently addressed the conclusion by Hutchison et al. (2008), that LSA cosine similarities do not predict priming effects at the item level in a long-SOA lexical decision priming paradigm (Günther et al., 2016), while following the reasoning just presented. To this end, we employed a technique to generate the item material where we pseudo-randomly generated word pairs while controlling their LSA cosine similarities: First, we selected a fixed set of words. Each of these words was then assigned a specific cosine similarity range (for example, if “house” was a word in the set, it could be assigned to a cosine similarity between 0.00 and 0.09). We then sampled all the words within this cosine similarity range to the target word, randomized their order, and selected the first word to meet some criteria regarding words lengths and frequencies as the second word of the pair. This technique has at least two advantages over *post-hoc* analyses: On the one hand, it ensures that any word pair can occur in the item material, and therefore prevents a biased selection of items (see Forster, 2000, for an illustration of how manually selecting items in language experiments can potentially influence the size of observed priming effects). The word pairs included in the study can therefore be seen as a random sample from the set of possible word pairs, given the pre-selected words (with some constraints, as described in the *Method* sections). On the other hand, it simultaneously ensures that the analyzed cosine similarities are systematically controlled for and evenly distributed, and that they can be analyzed as a numerical variable rather than a group variable. In our previous experiments, we found that LSA cosine similarities did indeed predict priming effects at the item level.

## Objectives for the current study

The present study is based on the study by Günther et al. (2016). It has two, related objectives: One purpose of the present study is to replicate the findings of Günther et al. (2016) that word similarities obtained from DSMs predict priming effects, and therefore can be assumed to reflect cognitive word similarities. The recent debate on the replicability of psychological research (Open Science Collaboration, 2015) has shown that replications are an important component of the scientific method, which help to reduce the impact of false-positive results (Ulrich et al., 2016) and to get a more precise picture of effect sizes associated to the respective phenomena.

Additionally, and more critically, we want to address several methodological issues of the study by Günther et al. (2016). Most importantly, we use a far larger corpus to construct our semantic spaces. It has been shown that larger corpora, and hence more data input, improve the stability of vector representations in DSMs, and therefore the quality of the obtained cosine similarities (Brill, 2003; Bullinaria and Levy, 2007, 2012). For the purposes of constructing a DSM model, the ~5 million word corpora used by Günther et al. were quite small, which might have lead to a lot of noise in the semantic spaces used there. Therefore, it is possible that similarity values for many word pairs have been over- or underestimated, which would directly result in suboptimal item material for this study, following from the item selection technique that was employed. This instability of the vector representations for the item material might therefore directly lead to an instability of the empirical results, since vector representations derived from a different corpus of the same size might be very different. In our view, this makes a follow-up study that is based on better vector representations (derived from more corpus data) necessary. In order to address this issue, we now constructed our semantic spaces from a corpus that was substantially larger (~880 million words, which is more within the dimensions of the current literature on DSMs). The selection of a larger corpus enabled another incremental improvement over our previous experiments: In Günther et al. (2016), word pairs with cosine similarities values over 0.5 could not be considered for the item material, since many words did not have any other words within this similarity range. Therefore, we were not able to analyse the full range of cosine similarity values in our previous study, which will be possible in the current study.

Further, extending the Günther et al. (2016) study, we did not only use an LSA-type space to generate material (Experiment 1), but also a HAL-type space (Experiment 2). Although we re-analyzed the data of our previous experiments with HAL similarities (Günther et al., 2016), this was done as a *post-hoc* analysis, and the rationale of constructing item material from a specific semantic space is to avoid such *post-hoc* analyses, as discussed earlier. Therefore, constructing item material directly from both an LSA-type space as well as an HAL-type space allows us to generalize our findings from only one specific model to other DSMs. This is especially interesting since LSA and HAL rely on fairly different input data, resulting in different representations, with the document-based LSA algorithm better suited to capture associative relations between words, and the word-based HAL algorithm more focussing on semantic relations. Employing both models to generate item material therefore allows us to more directly compare how both these models predict priming effects, and whether they perform differently.

As additional improvements over our previous study, we ensured that the distribution of cosine similarity values in our current study was much smoother and more uniform than in our previous study. Since we investigated cosine similarities a linear predictor in this study, ensuring a distribution of this parameter that is as uniform as possible leads to better and more precise estimations of the actual effect this parameter has on priming effects.

Taken together, there were several methodological issues in the Günther et al. (2016) study that can put the results published there in question. In order to address these issues and to further established the results obtained by Günther et al. (2016) with a more solid methodology, we set up the experiments presented in the current study with the aim to replicate those findings. For these experiments, we employed the same pseudo-random method for directly manipulating DSM cosines in generating item material that was introduced in Günther et al. (2016). Our hypothesis was that higher cosine similarities predict faster reaction times in a lexical priming paradigm with a lexical decision task, and hence that we find a negative relation between DSM cosine similarities and reaction times.

## Corpus and semantic spaces

As a source corpus to create the semantic spaces employed in this study, we used the ~880 million word sDeWaC corpus (Faaß and Eckart, 2013), which is organized into ~1.5 million documents. As can be seen, this corpus is considerably larger than the ~5 million word corpus employed in Günther et al. (2016). From this corpus, we created two different semantic spaces: A document-based LSA-type space, and a moving-window based HAL-type space. Both semantic spaces were created using the *DISSECT* toolkit (Dinu et al., 2013), and are freely available in the .rda format for R (Günther et al., 2015).

For the LSA-type space (from now on referred to as *LSA space*), we constructed a word × document co-occurrence matrix, where each cell entry specifies how often a word occurs in a given document. The rows of this matrix represent the 100,000 most frequent words in the corpus, and the columns represent the ~1.5 million documents. We applied a positive Pointwise Mutual Information (Church and Hanks, 1990) weighting on the raw co-occurrence counts, and reduced the matrix from 1.5 million to 300 column dimensions using Singular Value Decomposition (Martin and Berry, 2007).

For the HAL-type space (from now on referred to as *HAL space*), we constructed a word × word co-occurrence count matrix. Two words were considered to co-occur if both occurred within a window of three content words within a sentence. This window size differs from the 8-word window initially suggested by Lund and Burgess (1996), but it has been repeatedly shown that smaller window sizes of around 2–3 words produce better results than larger windows over a variety of tasks (Bullinaria and Levy, 2007, 2012). Converging results were obtained for semantic priming data, with window sizes of three words best predicting priming effects (Mandera et al., 2017)^1^. Only the 100,000 most frequent words in the corpus were considered as rows and columns of the matrix. As in the LSA space, a positive Pointwise Mutual Information weighting was applied, and the matrix was reduced from 100,000 to 300 dimensions using Singular Value Decomposition.

## Experiment 1

### Methods

#### Participants

All participants volunteered to participate in this experiment and gave informed consent. We tested 44 native German speaking participants (29 female, 15 male) for this experiment, with a mean age of 22.5 years (*SD* = 3.2 years). Of those, 13 were left-handed. Participants received either money or course credit for their participation.

#### Material

To create our material, we employed the pseudo-random item generation method introduced in Günther et al. (2016), with slight modifications.

First, we selected a set of 300 medium-frequency German words (frequency class between 10 and 15 according to http://wortschatz.uni-leipzig.de)^2^, which were concrete nouns with a length between 4 and 9 letters. Of these, 200 were randomly selected as target words, and the remaining 100 were used as primes for the nonword trials.

We then pseudo-randomly assigned a prime word to each target word, on the basis of LSA cosine similarities. We defined 10 similarity classes (the first class with similarities between 0.00 and 0.09, the second class between 0.09 and 0.18, …, the last class between 0.81 and 1), and randomly assigned 20 targets to each of these classes. This was done to ensure an approximately uniform distribution of cosine similarity values in our material. Contrary to the original study Günther et al. (2016), we used more similarity classes to ensure a more uniform cosine distribution, and covered a wider range of cosine values.

For each target word, we then randomly sampled a prime word which was represented in the LSA space, with the constraint that the cosine similarity between prime and target was in the similarity class assigned to the target. As further constraints, we selected only concrete nouns with a length between 4 and 10 letters and a frequency class between 9 and 16 (according to http://wortschatz.uni-leipzig.de) that were not yet part of the item material. Some targets had to be randomly re-assigned to another similarity class in cases were no word meeting all constraints could be found. For this selection procedure, we used the package *LSAfun* (Günther et al., 2015) for R (R Core Team, 2015).

As nonword targets, we constructed 100 nonwords with a length between 4 and 10 with a German phonetic and subsyllabic structure using *Wuggy* (Keuleers and Brysbaert, 2010). Nonwords and real target words did not differ significantly in length, and real target pairs and nonwords target pairs did not differ significantly in prime length and frequency. We further ensured that, for the real target pairs, LSA cosine similarities were not significantly correlated with target and prime lengths and frequencies. The item material for Experiment 1 can be found in Data Sheet 1.

The item material was randomly ordered into four item blocks, each containing 50 real target pairs and 25 nonword target pairs. We included fewer nonword than real target trials in order to keep the experiment short and to prevent possible effects of fatigue. A nonword ratio of less than 50% is not unusual in the priming literature (Chiarello and Richards, 1992; McNamara, 1992; Abernethy and Coney, 1996; Smolka et al., 2014, however, see the Discussion section for potential effects of the nonword ratio). Word stimuli were always presented in black letters in the center of a white screen. Capital letters had a height of 9 mm, with a width between 3 and 9 mm; lower case letters had a height of 7 mm, with a width between 2 and 9 mm. All words began with a capital letter, while the other letters were in lower case (the typical German orthography for nouns).

#### Procedure

At the beginning of the experiment, participants were instructed that they would be presented with word pairs, to which they had to react by pressing the right key (END on a standard keyboard) if the second word (i.e., the target) was a real word, and the left key (TAB on a standard keyboard) if it was not an existing German word. The response keys were marked with green stickers.

The trial procedure was identical to the original study (Günther et al., 2016). Each trial began with a 7 × 7 mm black fixation cross presented in the center of the screen, which was then followed by the prime word for 500 ms. After the prime word, a blank screen appeared for another 500 ms, and then the target word was presented. This procedure induces a relatively long stimulus onset asynchrony (SOA) of 1000 ms. We opted for a long SOA since typically sligthly larger priming effects are observed at longer SOAs (over 1000 ms) as compared to shorter SOAs (around 250 ms; de Groot, 1984; Neely, 1991; Hutchison, 2003; Hutchison et al., 2008), especially for associatively related word pairs. This finding was explained by assuming that a full processing of the prime is not completely finished at short SOAs (Neely and Keefe, 1989; Hutchison et al., 2008), and that associative priming might be—at least in part—driven by expectancies about the target word that are generated after the prime has been processed. In order to establish an experimental setting that was best suited to detect both kinds of priming effects—semantic and associative—we therefore opted for a long SOA, as both types of priming occur at long SOAs, while effects at short SOAs are less stable. However, the general presence or absence of priming effects in LDTs seem to be largely independent of SOA (Lucas, 2000), at least for the large majority of typical priming phenomena (Neely, 1991). The target word disappeared after a response was made (followed by either a *Richtig!* (*Correct!*) feedback in green letters, or a *Fehler!* (*Error!*) feedback in red letters for 1000 ms), or after 3000 ms, at which point *Zu langsam!* (*Too slow!*) appeared in red letters. After another blank screen for 500 ms, a new trial began.

The items were presented in four blocks, as described above. Therefore, each participant saw each item exactly once. The item order within the blocks was randomized; the order of the blocks was balanced over participants using a Latin square design. After each block, participants had the possibility to take a break. The actual experiment was preceded by a practice block containing 20 items of which no word appeared in the item list. The whole experiment took about 20 min to complete.

### Results

We analyzed only trials in which the target was a real word. No participants or items had to be removed due to high error rates (we set the exclusion criterion for both items and participants to error rates >25%). We excluded trials in which an erroneous answer was made (1.7%), as well as trials with reaction times (RTs) under 100 ms or over 1500 ms (0.4%). As in the original article (Günther et al., 2016), we analyzed logarithmic reaction times (logRTs) instead of raw RTs (Baayen and Milin, 2015), since their distribution was far closer to a normal distribution.

We employed Linear Mixed Effect Models (LMEMs) to analyse our data (Baayen et al., 2008), using the package *lme4* (Bates et al., 2015) for R (R Core Team, 2015). To test for effects of cosine similarities on logRTs, we performed likelihood-ratio tests. Our baseline models hereby contained fixed effects for prime and target lengths and frequencies, random intercepts for subjects and items, as well as by-subject random slopes for target lengths and frequencies as well as the cosine similarity in question, following Barr et al. (2013). Contrary to Günther et al. (2016), we included by-subject random slopes for cosines *before* testing for cosine fixed effects, which is more in line with the actual suggestions by Barr et al. (2013). By-subject random slopes for prime lengths and frequencies were not included since these variables had little to no predictive power as fixed effects, and some models did not converge when containing these effects.

In R syntax, the baseline models looked as follows:

logRT ~ LengthTarget + FrequencyTarget 
        + LengthPrime + FrequencyPrime
        + (Cosine + LengthTarget +
        FrequencyTarget | Subject)
        + (1 | Item)


We then additionally added a fixed effect parameter for cosine similarities to those models, and compared the two models using likelihood-ratio tests.

**Figure 1:**
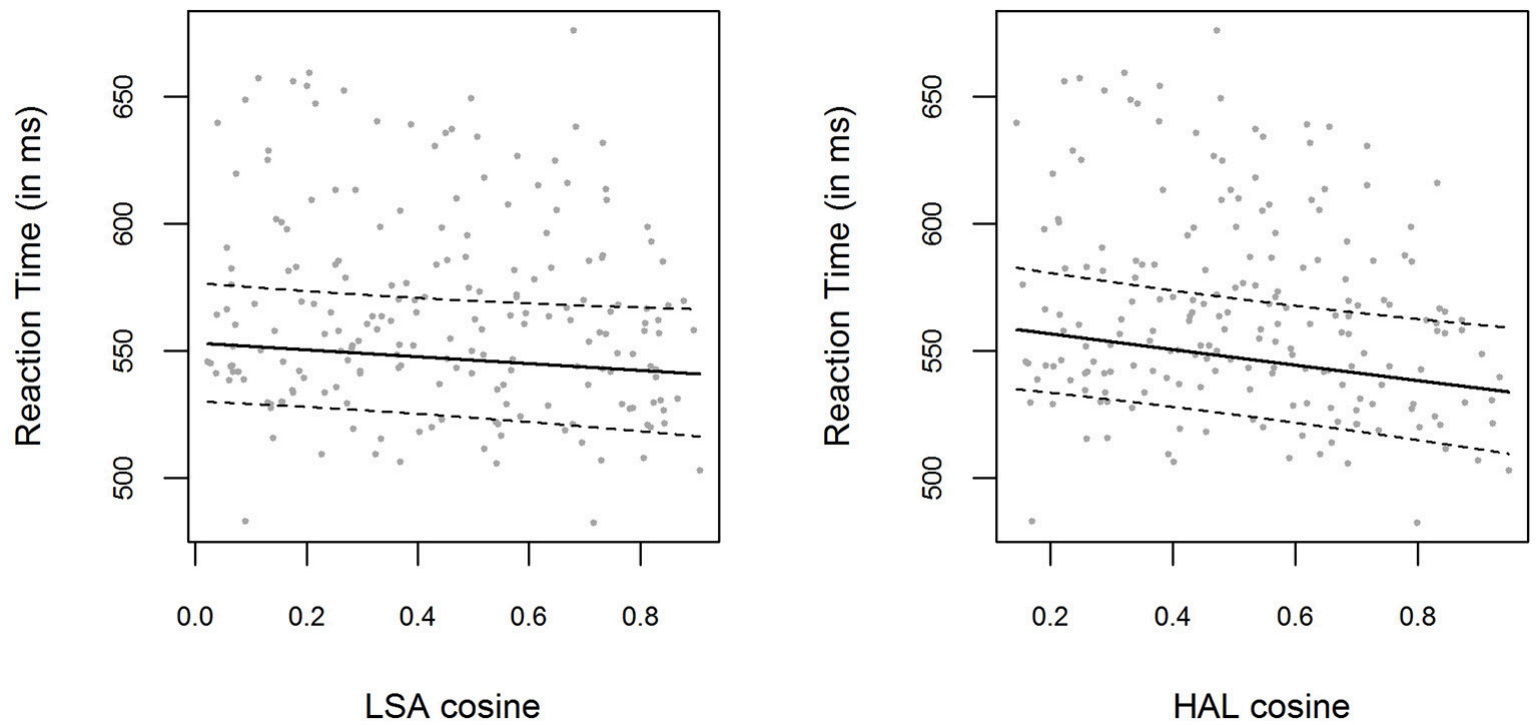
**(Left panel)** Mean RTs for each item in Experiment 1, plotted as a function of the prime-target LSA cosine similarity. The solid line shows the exponentiated model predictions of the model including cosine fixed effects, with the corresponding 0.95 confidence interval (dashed line). **(Right panel)** Mean RTs and model predictions, as in the left panel, for HAL cosine similarities.

The bivariate relations between LSA and HAL cosine similarities and logRTs are displayed in Figure 1, with model predictions obtained with the *effects* package (Fox, 2003) for R. Including a fixed effect for the LSA cosine similarities (which were used to create the material for this experiment) did not significantly improve the baseline model [$\chi_{\left(1\right)}^{2}=2.27,$ *p* = 0.132]. The parameter estimate for the LSA cosine similarity parameter was β = −0.025 (*t* = −1.51), with a corresponding 0.95-Wald-CI^3^ of [−0.057, 0.007], containing zero. However, performing the same analysis using the HAL cosine similarities instead of LSA cosine similarities resulted in a significant improvement of the model predictions [$\chi_{\left(1\right)}^{2}=8.15,$ *p* = 0.004]. The parameter estimate for the HAL cosine similarity parameter was β = −0.056 (*t* = −2.88), with the 0.95-Wald-CI being [−0.094, −0.018]. The bivariate correlation between LSA cosines and HAL cosines was very high, *r* = 0.89.

## Experiment 2

In Experiment 1, the item material was generated based on LSA cosine similarities, and analyzed using LSA and HAL cosine similarities. To get a more comprehensive view of the effects these two variables have on priming RTs, we also generated another item set based on HAL cosines.

### Methods

#### Participants

All participants volunteered to participate in this experiment and gave informed consent. For this experiment, we tested 43 native German speaking participants (25 female, 18 male), with a mean age of 24.44 years (*SD* = 5.7 years). Of those, 12 were left-handed. Participants received either money or course credit for their participation. No participant took part in both Experiment 1 and Experiment 2.

#### Material and procedure

The material was constructed using the same procedure as described in Experiment 1. The same real word targets as in Experiment 1 were used, as well as the same primes for nonword trials. Prime words for the real word targets were again selected employing the same pseudo-random selection procedure, with the major difference being that HAL cosine similarities were used to generate the material instead of LSA cosine similarities. The constraints on potential prime words were the same as in Experiment 1 (concrete noun, length between 4 and 10 letters, frequency class between 9 and 16).

We also generated new nonword targets using *Wuggy* (Keuleers and Brysbaert, 2010). Therefore, there was no item that occurred both in Experiment 1 and Experiment 2. Again, nonwords and real target words did not differ significantly in length, real target pairs and nonwords target pairs did not differ significantly in prime length and frequency, and HAL cosine similarities were not significantly correlated with target and prime lengths and frequencies. The item material for Experiment 2 can be found in Data Sheet 2.

The procedure of Experiment 2 was identical to Experiment 1.

### Results

Three participants had to be excluded due to overall high error rates (>25%). We further excluded 1.8% of the trials due to erroneous answers, and of the remaining trials we excluded another 0.3% due to RTs under 100 ms or over 1500 ms.

**Figure 2:**
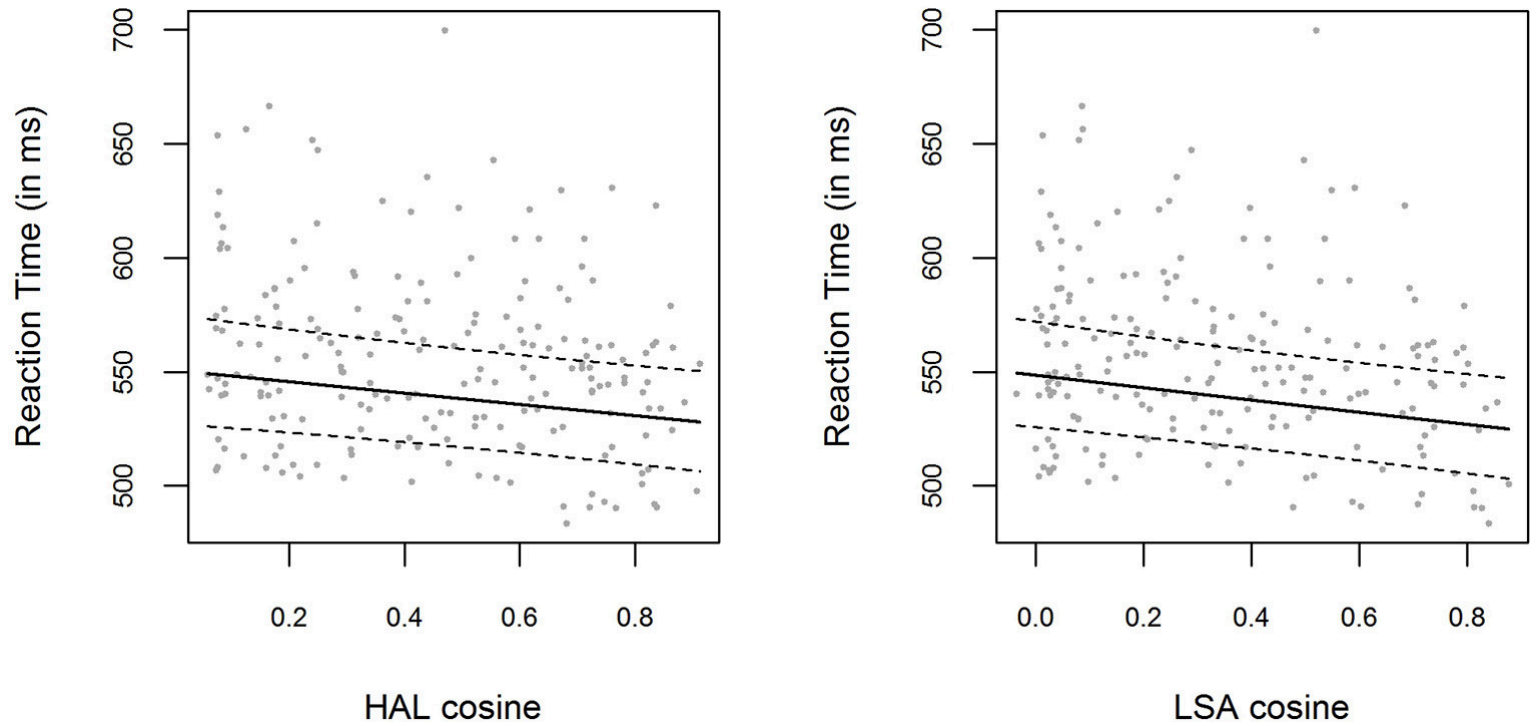
**(Left panel)** Mean RTs for each item in Experiment 2, plotted as a function of the prime-target HAL cosine similarity. The solid line shows the exponentiated model predictions of the model including cosine fixed effects, with the corresponding 0.95 confidence interval (dashed line). **(Right panel)** Mean RTs and model predictions, as in the left panel, for LSA cosine similarities.

The bivariate relations between LSA and HAL cosine similarities and logRTs are displayed in Figure 2. We employed the same LMEM analysis as described in the *Results* section of Experiment 1. Including a fixed effect for the HAL cosine similarities (which were used to create the material for this experiment) significantly improved the baseline model [$\chi_{\left(1\right)}^{2}=7.90,$ *p* = 0.005]. The parameter estimate for the HAL cosine fixed effect was β = −0.046 (*t* = −2.84), with the 0.95-Wald-CI being [−0.078, −0.014]. The same pattern emerged if we performed the same analysis with LSA cosine similarities instead of HAL cosine similarities: Including LSA cosines in the model significantly improved model predictions [$\chi_{\left(1\right)}^{2}=9.75,$ *p* = 0.002]. The parameter estimate for the LSA cosine fixed effect was β = −0.050 (*t* = −3.22), with the 0.95-Wald-CI being [−0.081, −0.020]. Again, the correlation between HAL cosines and LSA cosines was very high, *r* = 0.93

#### Analysis of pooled data

In order to get a more reliable estimate of the effects of LSA and HAL cosine similarities and a more comprehensive view on our data, we pooled the data from both experiments, and conducted a single analysis on this pooled data. Note that this analysis is a *post-hoc* analysis over different experiments.

The model comparisons we performed were essentially the same as in the analyses reported above; however, since all target words appeared both in Experiment 1 and Experiment 2 (with different prime words, respectively), we included random intercepts for primes as well as targets instead of just random intercepts for items in our models^4^. Although justified by the structure of the pooled data, we did not include any by-target random slopes, due to convergence issues.

**Figure 3:**
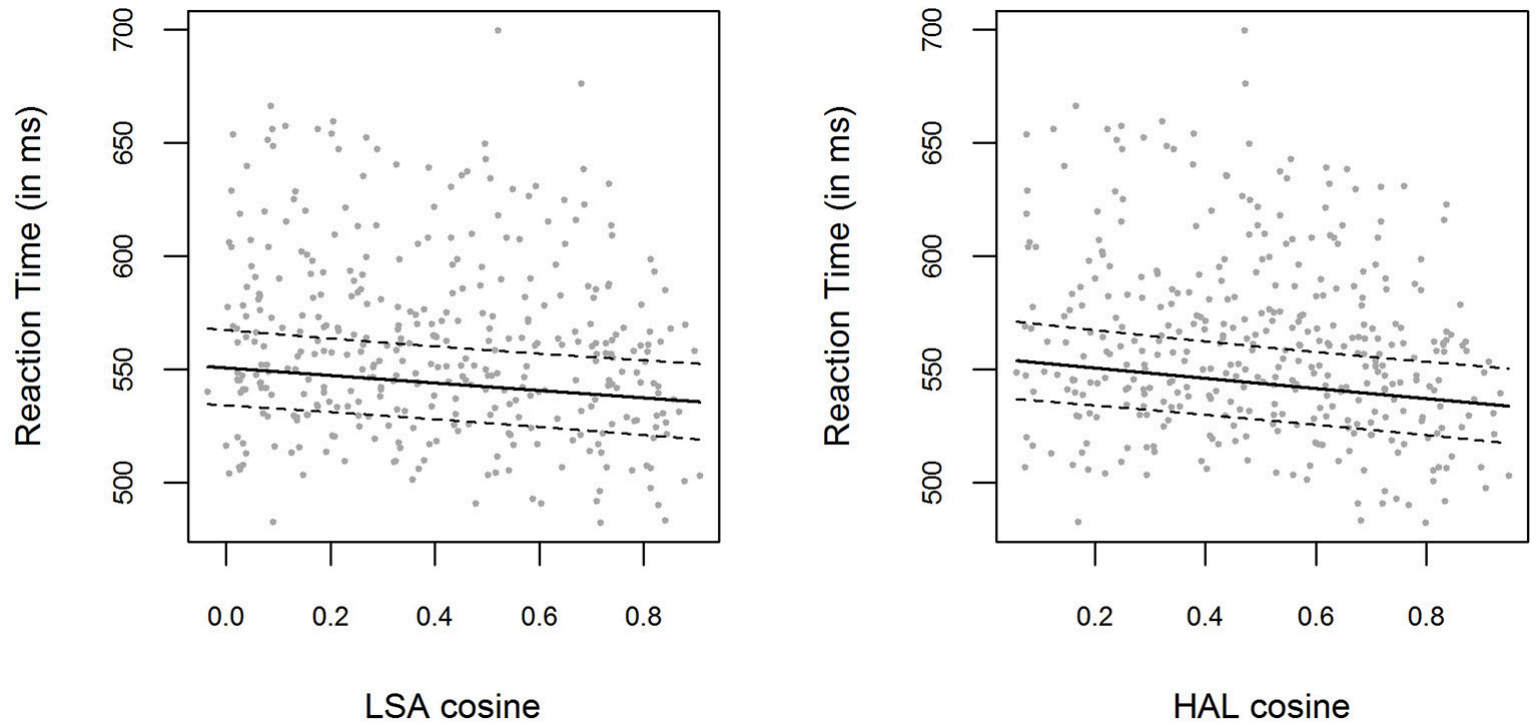
**(Left panel)** Mean RTs for each item in the pooled data from Experiment 1 and Experiment 2, plotted as a function of the prime-target LSA cosine similarity. The solid line shows the exponentiated model predictions of the model including cosine fixed effects, with the corresponding 0.95 confidence interval (dashed line). **(Right panel)** Mean RTs and model predictions, as in the left panel, for HAL cosine similarities.

The bivariate relations between LSA and HAL cosine similarities and logRTs for the pooled data are displayed in Figure 3. Including a fixed effect for LSA cosine similarities significantly improved the respective baseline model [$\chi_{\left(1\right)}^{2}=10.29,$ *p* = 0.001]. The parameter estimate for the LSA cosine fixed effect for the pooled data was β = −0.028 (*t* = −3.24), with the 0.95-Wald-CI being [−0.045, −0.011]. On the other hand, including HAL cosines in the model also results in a model that outperforms the baseline model [$\chi_{\left(1\right)}^{2}=15.94,$ *p* < 0.001]. The parameter estimate for the HAL cosine fixed effect was β = −0.039 (*t* = −4.03), with the 0.95-Wald-CI being [−0.057, −0.020]. The correlation between HAL cosines and LSA cosines for all the item pairs in the pooled data was *r* = 0.91.

The measures for model fit can be compared, since both models are estimated on exactly the same data. As can be seen in Figure 3, the LSA and HAL cosines cover the same range of values, and can therefore also be compared in a meaningful fashion (this point is illustrated in the *Discussion*). The results indicate that the effect of HAL cosine similarities is more prominent than the effect of LSA cosine similarities: First of all, the parameter estimate for HAL cosines is higher (more specifically, more negative) than that for LSA cosines, with the respective 0.95-Wald-CIs being of almost the same width. This is also reflected in the higher *t*-value for HAL cosines. Furthermore, including HAL cosine fixed effects improves the baseline model more than including LSA cosines does. Apart from a higher χ^2^-value in the likelihood-ratio test, this is also shown in higher log-likelihoods and lower AIC^5^ values for the model containing HAL cosines (*log* (*L*) = 3425, *AIC* = −6812) as compared to the model containing LSA cosines (*log* (*L*) = 3422, *AIC* = −6807).

#### Comparison with the original LSA space

We additionally tested whether the semantic spaces in the present study, created from ~880 million word corpora, indeed predicted reaction times better than the LSA space created from the 5 million word corpus employed in Günther et al. (2016). To this end, we re-analyzed the pooled data from both experiments. Not all words included in the item material of the present study had a vector representation in this smaller LSA space (which we will refer to as the *Blogs* space, since it was created from blog entries), and we excluded the respective 26 items from the analysis. Therefore, the results for the LSA and HAL space described earlier will be slightly different.

**Figure 4:**
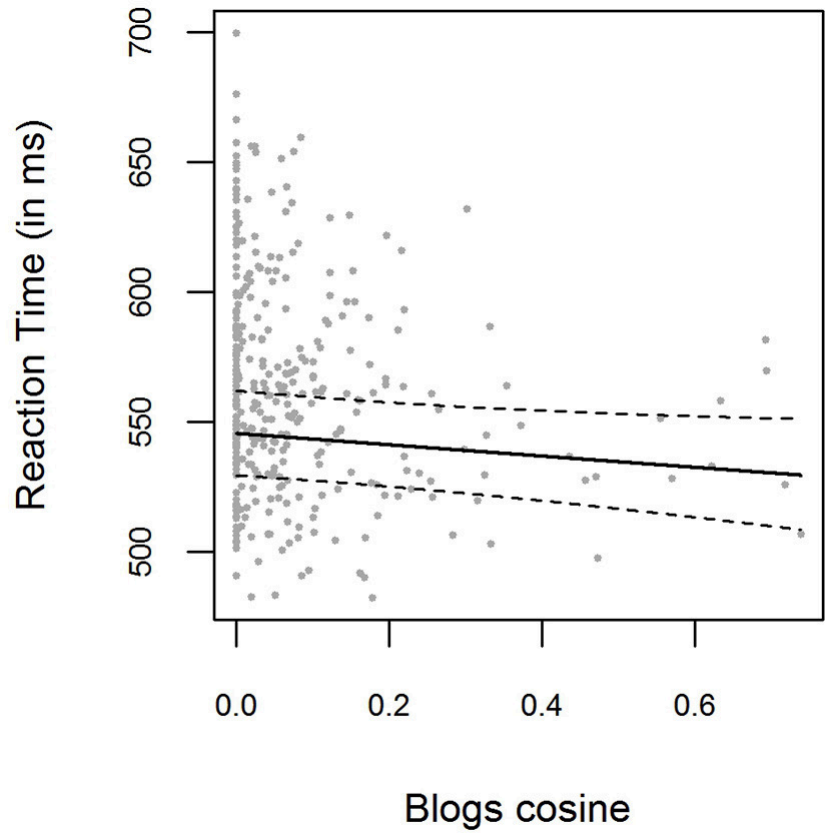
**Mean RTs for each item in the pooled data from Experiment 1 and Experiment 2, plotted as a function of the prime-target Blogs cosine similarity**. The solid line shows the exponentiated model predictions of the model including cosine fixed effects, with the corresponding 0.95 confidence interval (dashed line).

Negative Blogs cosine values were set to zero. The bivariate relations between Blogs cosine similarities and logRTs for the pooled data are displayed in Figure 4. Blogs cosine similarities were not found to significantly improve the baseline model [$\chi_{\left(1\right)}^{2}=3.40,$ *p* = 0.065]. The parameter estimate for the Blogs cosine fixed effect was β = −0.040 (*t* = −1.85), with the 0.95-Wald-CI being [−0.083, 0.002]. For the smaller item set analyzed here, LSA cosines improved baseline model predictions [$\chi_{\left(1\right)}^{2}=10.81$, *p* = 0.001, β = −0.30, *t* = −3.33, *CI* = [−0.048, −0.012]], as did HAL cosine similarities [$\chi_{\left(1\right)}^{2}=16.16$, *p* < 0.001, β = −0.041, *t* = −4.08, *CI* = [−0.061, −0.022]]. The correlation between Blogs cosines and LSA cosines was 0.41, the correlation between Blogs cosines and HAL cosines 0.39.

For the model including Blogs cosines, the model log-likelihood was lower (*log* (*L*) = 3126) than for the LSA model (*log* (*L*) = 3131) or the HAL model (*log* (*L*) = 3134), and the AIC was higher (*AIC* = −6215) than in the LSA model (*AIC* = −6223) or the HAL model (*AIC* = −6229). As can be seen, both the LSA and HAL cosines outperform the Blogs cosines as a predictor of reaction times.

## Discussion

In the two experiments reported above, we examined whether DSM similarity measures—more specifically, HAL and LSA cosine similarities—predict priming effects at the item level, in order to replicate and extend the findings of Günther et al. (2016). To this end, we employed the pseudo-random technique to generate item material introduced by Günther et al., which enables an unbiased selection of the item material on the basis of cosine similarities, while at the same time ensuring an even distribution of these similarities. Contrary to Günther et al. (2016), we used a far larger corpus to generate the semantic spaces, also used a HAL space to generate item material, examined a wider range of cosine similarities with a more uniform distribution, and better adjusted our data analysis to the actual experimental design. Furthermore, in each of the experiments we used new word pairs, thereby generalizing the results to other item material.

In Experiment 1, where the items were generated using LSA cosine similarities, we did not find that LSA cosines predicted priming effects, while HAL cosines did. In Experiment 2, where HAL cosine similarities were used to create the material, we found that both LSA cosines and HAL cosines predicted priming effects. In our analysis of the pooled data of Experiment 1 and Experiment 2, both similarity measures were found to predict priming effects. Interestingly, the correlations between the two similarity measures were very high in both studies. Note that, although in each of the experiments one of the cosine similarities was only analyzed as a *post-hoc* variable, they still showed the properties intended with the pseudo-random item generation: The item pairs were constructed by random sampling and therefore cannot be the results of a biased selection, and the distribution of both LSA and HAL similarities was very even in both experiments.

Taken together, we take our results as a confirmation of our hypothesis that priming effects can be predicted from DSM similarity measures when the item material is directly generated from these models and the DSM similarity measures are directly manipulated as an independent variable. This extends insights from *post-hoc* analyses of existing priming effects using DSM measures: It addresses the question whether differences in DSM similarities between word pairs are sufficient to produce priming effects, and provides positive evidence in this direction.

With these results, we replicate the pattern of results obtained by Günther et al. (2016). Note that, at the first sight, the parameter estimates for the Blogs cosine similarities seem to be higher in the study by Günther et al. than they are for the LSA and HAL space in the present study (β-weights had values of around −0.05 to −0.06, compared to the estimates around −0.03 to −0.04 found here). However, this is an artifact caused by the quality differences of the semantic spaces that were taken to analyse the data. In the Blogs space used by Günther et al., high cosine similarities were extremely rare (as illustrated in the analysis with the Blogs space in this study and in Figure 4), and they basically capped at 0.6, which was therefore set as the highest cosine value in the item selection procedure. The LSA and HAL spaces used in the present study however produced cosine similarities covering the full range up to 1.00. Therefore, if the highest cosine similarity one can reasonably achieve is 0.6 in the one and 1 in the other space, it follows naturally that the β-weight (which indicates what change in logRT coincides with a change of 1 in the respective predictor) is higher in the first case, given the reaction time pattern is the same for both cases. Additionally, the confidence intervals for parameter effects were far narrower for the LSA and HAL space used in the present study. The fact that the parameter estimate for the Blogs space in this study is lower than in the original study, indeed to such an extent that we find no significant effect for this parameter in the present study, is likely caused by the fact that the Blogs cosine distribution is very skewed here, with most word pairs showing similarities around zero.

### On the differences between HAL and LSA

Given the results we just described, how do we interpret the absence of a significant effect of LSA cosine similarities of Experiment 1? We propose to take this as an informative piece of evidence, which suggests that HAL cosines are a better predictor for priming effects than LSA cosines for our data. This interpretation is supported by the results we obtained in the pooled analysis (smaller parameter estimate with a comparable confidence interval width, the LSA model had a lower model likelihood and a higher AIC).

This raises the question why HAL similarities emerge as a better predictor in our experiments than LSA similarities. One possible explanation can be given by considering the different types of information that are encoded in the vector representations of the different models, and that ultimately determine the similarity values. HAL vector representations are derived from a word × word co-occurrence count matrix. Since we employed a three-word window, this means that words with a very similar immediate surrounding will have similar vector representations. Hence, the HAL algorithm (given the co-occurrence window is not too large) focusses on local information on words, which might make it more suitable for predicting word-word priming effects: Words that are very similar in terms of HAL similarity scores occur in very similar word context, and can therefore be very likely substituted by one another (i.e., they stand in a paradigmatic relation, as discussed in the introduction). This ultimately means that they most likely denote very similar semantic concepts, which gives rise to the prediction of priming effects observed here.

The LSA vector representations, on the other hand, are derived from word × document co-occurrence counts. Therefore, words with a similar distribution over a range of documents will have similar vector representations. However, this does not include much information about the direct relation between the words themselves; it rather indicates that they play a role for similar topics (Griffiths et al., 2007). This is a rather global information about the role of words, when compared to the local context information that the HAL algorithm relies on. While LSA captures global word information across documents, HAL is much more sensitive toward the immediate, local context of the word, which might give rise to the differences in predictive power for the priming effects observed in our word-to-word priming experiments.

This reasoning converges with more general findings that semantic priming effects between words are generally stronger than priming effects due to purely associative relations (Lund et al., 1995, 1996; Jones et al., 2006). In an experiment using word pairs that were either purely semantically related, purely associatively related, or both, using item material from a study by Chiarello et al. (1990), Lund et al. (1995) observed no priming effects for the purely associatively related pairs. From this, Lund et al. (1995) initially concluded that there is no reliable effect of associative relations, if semantic relations between the respective words are controlled for. They argued that associative priming effects found in earlier studies are not actually due to associative relations, but rather to the fact that there were also semantic relations for the associatively related word pairs used in these studies (such as *road*-*street* or *girl* - *boy*). However, later studies showed that priming does indeed occur for purely associatively related word pairs with carefully controlled material (Lund et al., 1996; Ferrand and New, 2003), but that associative priming effects are smaller in size and less reliable (Lund et al., 1996).

Note that, following from the method used to create the item material in our study, it is highly unlikely that many word pairs in the material share “purely semantic” or “purely associative” relations (for example, a word pair with high a HAL similarity (>0.8) in our item material is *Flöte - Posaune* (*flute - trombone*), which clearly is related associatively as well as semantically). Instead, most items share both semantic and associative relations, both to some degree. Such a blend of associative and semantic relations is presumably given for most word pairs (Lund et al., 1995, 1996; Hutchison, 2003). Therefore, we assume in our argumentation that, in general, both semantic and associative take place for a given word pair, depending on how strong the respective relations are for that pair. That both types of priming can occur simultaneously is illustrated, for example, by the finding that there is an associative boost in priming (Moss et al., 1994; Lucas, 2000; Jones et al., 2006): Pairs that are associatively as well as semantically related show stronger priming effects than purely semantically related pairs.

Following up on the assumption that HAL similarities predict the semantic parts of priming, and LSA similarities predict the associative parts of priming, our results that HAL similarities are the stronger predictor lead us to the following conclusion: Whether two words denote a similar concept seems to influence the (psychological) similarity between the words, as reflected in priming effects, more than whether the two words (or the concepts they denote) regularly occur together.

However, one drawback of the present experiments is that in each experiment, only one DSM similarity measure was manipulated. In order to more rigorously investigate the assumptions made here, it would be desirable to set up experiments where both HAL and LSA similarities (that is, presumably, semantic and associative similarities) are manipulated orthogonally at the same time. This would allow to more strictly compare their effects in a single experiments, and to investigate phenomena such as associative boost in more detail.

### On the experimental setting—SOA and nonword ratio

In the experiments reported here, we made choices regarding the parametrization of the experimental procedure that can be argued to increase the likelihood that participants developed strategies or expectancies which affected the results.

Firstly, we opted for a very long SOA of 1000 ms, which included an inter-stimulus interval of 500 ms. This gave the participants a long time to process the prime stimulus, and to generate expectancies for possible target stimuli. This is indeed assumed to be one of the main reasons why priming effects are more prominent for long SOAs (Becker, 1980; Neely et al., 1989): When participants have a lot of time to process a prime word, they generate a set of likely candidates that might follow the prime. If the actual target then is part of this candidate set, the lexical access is facilitated and response times are faster.

Secondly, participants were presented with twice as many real word target trials than nonword trials. This might have biased the participants toward responding with a “yes” in the lexical decision task. Apart from possibly causing faster “yes” than “no” responses (which would not concern us since we did not analyse “no” responses), it has also been shown that the nonword ratio affects priming effects, in that priming effects are stronger the fewer nonwords are presented (Neely et al., 1989). This can be explained by assuming a semantic matching process between the prime and the target, which biases responses toward “yes” (i.e., the target is a word) for targets which are highly related to the prime, and toward “no” (i.e., the target is not a word) for more unrelated targets (Neely et al., 1989). The fewer actual nonwords are present in an experiment, the more are responses biased toward a “no” response in general. This makes the tendency toward a “no” response for unrelated targets harder to overcome, which slows down response times for these targets and increases priming effects.

Taken together, given our experimental setup, it is quite possible that strategies were employed by the participants and that expectancies were generated. Therefore, cannot derive statements about automatic processes from our results. However, even when assuming that expectancies and strategies play a role, our results still speak in favor of the psychological validity of DSMs: To generate expectancies about possible targets that follow a prime, the participant has to rely on some knowledge about which targets are related to the prime. If DSMs correctly predict these candidates and to capture this knowledge, they have psychological validity. Furthermore, the discussion on expectancies and response biases for the nonword ratio also depends on the notion of more or less related words. If DSMs can capture which words are more and which are less related, this again speaks in favor of our hypothesis that similarity measures derived from DSMs do reflect psychological similarities between words.

This leaves open the question of whether one would expect similar results as the ones observed here in a different experimental setting in which expectancies and strategies should plays less of a role. In her meta-analysis on different studies of semantic priming, Lucas (2000) found that the size of pure semantic priming did not depend on SOAs. A similar pattern of results was found for the nonword ratio. From these findings, Lucas (2000) concluded that pure semantic priming effects are not expectancy-based but rather automatic. Therefore, by assuming that HAL similarities capture semantic relations between words, we would assume to observe similar effects of HAL similarities in a setting with short SOAs and/ or more nonwords. This also is in line with the claim by Burgess and Lund (2000), that HAL similarities indicate which information is activated in an automatic, bottom-up process upon encountering a prime word, which should be independent of SOA.

As for associative priming effects, it has been found that these are stronger for longer SOAs (Hutchison, 2003; Hutchison et al., 2008), and to be more expectancy-based than semantic priming effects. We therefore expect the predictive power of LSA cosine similarities to be weaker in a paradigm with shorter SOAs or more nonwords, but we do not expect it to disappear^6^. However, these questions ultimately have to be addressed in an empirical study.

### Further discussion

The purpose of the present study was to replicate findings from a previous study Günther et al. (2016), while also optimizing the methods used therein. To this end, we employed LSA and HAL cosine similarity measures as our variable of interest. As mentioned in the introduction of this article, a variety of different DSMs have been proposed, and we focussed only on HAL and LSA. We argue that, in order to examine whether other DSMs also predict priming effects, material should be generated from these models directly. Analyses of the present data sets with similarity values from other DSMs would be *post-hoc* analyses, which is what we wanted to avoid in the first place with our method of generating the item material from a specific model. The purpose of this study was therefore not to compare a variety of available models in terms of their predictive power for priming effects, which is an exploratory approach by nature, but to test a hypothesis about a specific relation between DSM similarities and behavioral effects in a confirmatory manner. A similar argument holds for analyses that explore which parameter set within the LSA and HAL model we employed best predict the priming effects. We believe that, in order to conduct such comparisons, a suitable way is not to rely on the data from single, standard-scale experiments, but instead to explore huge data sets of semantic priming. Efforts in this direction have very recently been made by Mandera et al. (2017), who relied on a large data set from Hutchison et al. (2013).

Given our results, we can conclude that distributional semantic similarity measures can in principle be assumed to reflect cognitive word similarities in humans, to at least such a degree that it can be observed in behavioral data. Furthermore, based on our results, we conclude that HAL cosine similarities are a more useful variable when it comes to predicting priming effects, a pattern which also emerged in *post-hoc* analyses conducted by Günther et al. (2016). Since HAL cosines capture semantic relations better while LSA cosines capture associative relations better (Jones et al., 2006; Sahlgren, 2008), these results are in line with findings that semantic priming effects are generally more prominent than associative priming effects Lucas (2000); Hutchison (2003). Our results further show that constructing semantic spaces from larger text corpora gives better vector representations (Brill, 2003; Bullinaria and Levy, 2007), which are more successful in predicting behavioral data.

Since the present study was conducted with German speaking participants, we used German language material, generated from German semantic spaces. However, we would expect similar effects to emerge in other languages, as already suggested by studies using English material (Jones et al., 2006; Hutchison et al., 2008). Further studies based on semantic spaces in other languages would be important to generalize DSM similarity measures as representing cognitive word similarities.

### Conclusion

The present results indicate that DSM similarity measures predict lexical priming effects and human behavioral data, and thus encourage the use of these measures of word similarity in psychological theories and empirical studies. They further qualify DSMs as being psychologically adequate and plausible. This allows for a transfer between computational linguistics and cognitive sciences, which proves to be fruitful for both fields (Lenci, 2008; Andrews et al., 2009; Baroni et al., 2010; Van Petten, 2014; Marelli and Baroni, 2015).

## Compliance with ethical standards

The experimental testing was in agreement with the guidelines for good scientific practice at the University of Tübingen (Germany). This was approved and checked by the Head of Psychology, Faculty of Science, University of Tübingen. Participants' anonymity was always preserved; at no point could the recorded data be associated with a participant's name. All participants provided written informed consent.

## Author contributions

Planning and Designing of the Experiments: FG, CD, BK. Conducting and Analyzing the Experiments: FG. Writing the Paper: FG, CD, BK.

## Funding

This project was funded by the German Research Foundation (DFG), Collaborative Research Centre 833 (SFB 833) “The Construction of Meaning”/Z2 project appointed to BK and Sigrid Beck. We acknowledge support for covering the publication fees by Deutsche Forschungsgemeinschaft and Open Access Publishing Fund of University of Tübingen.

### Conflict of interest statement

The authors declare that the research was conducted in the absence of any commercial or financial relationships that could be construed as a potential conflict of interest.

## Footnotes

^1^Note that window size might also be a factor influencing which kind of information other than semantic information is included in a word vector. For example, vectors built from smaller window sizes are supposed to capture more grammatical and syntactical information (for example, prepositions are often directly preceded by verbs). For more discussion on this topic, see Burgess and Lund (1997), Burgess et al. (1998), and compare also Elman (1990).

^2^With a frequency class of 10, the most common word in the source corpus (*der*) appears 2^10^ times as often as the word in question.

^3^Wald-CIs are computed from the parameter estimate β and the corresponding standard error.

^4^In Experiment 1 and 2, prime-target pairs were fixed, since each target was always presented with exactly one prime word. Hence, it made no sense to include both prime and target random effects there, since the two effects could not be estimated independently.

^5^The *Akaike Information Criterion* (AIC; Akaike, 1998) is a measure that captures both a model's complexity in terms of number of parameters and a model fit. Given two models describing the same data set, the model with a lower AIC value describes the data more efficiently.

^6^Note that Hutchison et al. (2008) initially predicted *stronger* effects of LSA cosine similarities at shorter SOAs, since they suspected that it captured automatic, contextual associations. However, these authors found no influence of LSA cosine similarities at long or at short SOAs, and no difference in the predictive power of LSA between those groups.
